# Engineering human Tregs to resist tacrolimus via FKBP12 gene editing

**DOI:** 10.3389/fimmu.2026.1756624

**Published:** 2026-04-17

**Authors:** Christopher J. Requejo Cier, Nicolas Valentini, Gabrielle Boudreau, Jean-Sébastien Delisle, Caroline Lamarche

**Affiliations:** 1 Centre de recherche de l’Hôpital Maisonneuve-Rosemont, Department of Microbiology, Infectiology and Immunology, Université de Montréal, Montreal, QC, Canada; 2 Centre de recherche de l’Hôpital Maisonneuve-Rosemont, Montreal, QC, Canada; 3 Centre de recherche de l’Hôpital Maisonneuve-Rosemont, Department of Medicine, Université de Montréal, Montreal, QC, Canada

**Keywords:** CRISPR-Cas9, FKBP12, immunotherapy, regulatory T cells (Tregs), tacrolimus

## Abstract

Regulatory T cells (Tregs) are essential for immune tolerance and are under active development as cell therapy in transplantation. However, the widespread use of the calcineurin inhibitor tacrolimus may inadvertently suppress Treg proliferation and activation, undermining their therapeutic potential. Tacrolimus binds to the FKBP12 protein in T cells, forming a complex that blocks calcineurin–NFAT signaling and suppresses IL-2 gene transcription, thereby inhibiting T cell activation. In this study, we investigated whether deleting FKBP12 in human Tregs could prevent tacrolimus-mediated suppression. Using CRISPR-Cas9 gene editing, FKBP12 was knocked out in *ex vivo* expanded human Tregs, which were then cultured for seven days with tacrolimus (10 ng/mL) or control, under varying IL-2 concentrations (100–500 IU/mL). We observed that tacrolimus significantly reduced the proliferation of control Tregs, even in conditions with 500 IU/mL IL-2, whereas FKBP12-knockout Tregs maintained robust proliferation comparable to untreated cells. We found no discernible changes in Treg phenotype or stability following FKBP12 deletion or tacrolimus exposure: edited Tregs retained normal expression of the lineage-defining marker FOXP3, displayed a global transcriptomic profile nearly indistinguishable from controls, and were similarly suppressive, indicating that they remained *bona fide* Tregs. These findings demonstrate that the antiproliferative effect of tacrolimus on Tregs is critically dependent on FKBP12, mirroring its mechanism in conventional T cells. By genetically uncoupling tacrolimus from its target in Tregs, this approach suggests a strategy to preserve Treg numbers during tacrolimus-based immunosuppression in transplant recipients, potentially enhancing Treg-based therapies for transplantation tolerance.

## 1 Introduction

Organ transplantation remains the only effective treatment for many end-stage diseases (1). Despite major clinical advances, alloimmune rejection still presents a significant challenge, requiring lifelong immunosuppression. Calcineurin inhibitors (CNI), especially tacrolimus, anti-metabolites and corticosteroids are commonly used to prevent graft rejection (2). However, long-term use of immunosuppressive drugs is linked to serious side effects, such as opportunistic infections, cancer, metabolic issues, and chronic graft dysfunction (3). These challenges have led to the exploration of alternative strategies to achieve lasting immunological tolerance.

Tacrolimus exerts its immunosuppressive effect by binding to the cytosolic protein FKBP12, forming a complex that inhibits the phosphatase activity of calcineurin (4). This inhibition prevents the dephosphorylation of the nuclear factor of activated T cells (NFAT) (4), thereby blocking its nuclear translocation and the transcription of IL-2 and other activation genes. As a result, T cell activation and proliferation are broadly suppressed, leading to a general dampening of immune responses (5).

Regulatory CD4^+^ T cells (Tregs), characterized by high expression of the transcription factor FOXP3 and IL-2 receptor (CD25) (6), have shown strong immunomodulatory abilities in transplantation contexts (7). Adoptive Treg therapy has become a promising strategy to promote graft tolerance (8–10) and lessen dependence on immunosuppressive drugs. However, these cell therapies are usually given alongside CNI, whose increased levels could probably also impair Tregs, including those expanded *ex vivo* and infused (11, 12).

There is an ongoing debate about whether tacrolimus suppress the activity of Tregs. Some studies suggest that Tregs possess distinct signaling pathways that make them inherently more resistant to CNI. For example, Tregs maintain a higher baseline level of nuclear NFAT (13–15), indicating that they continue to drive NFAT-mediated gene expression despite calcineurin blockade. This constitutive nuclear localization could make Tregs less dependent on new NFAT translocation events and may potentially preserve their activation potential when CNI are present. Collectively, this aligns with the idea that *in vitro* studies have reported that tacrolimus preferentially inhibits the proliferation of conventional T cells (Tconv), while relatively sparing Tregs. As a result, there is possibly a range of concentrations in which Tregs can expand while effector T cells are suppressed (16). In contrast, mounting data suggests that CNI may impede Treg activation and suppressive function in both animal and human subjects. In mice, researchers found that long-term tacrolimus treatment reduced Treg numbers and impaired their suppressive capacity. However, this effect could be reversed by the administration of exogenous IL-2 (13, 17). Individuals and nonhuman primates who receive long-term CNI therapy as part of their transplant treatment have been observed to have decreased circulating Treg numbers and suppressive function (11, 18, 19). The impact of tacrolimus on the stability, phenotype, and expansion of human *ex vivo* expanded Tregs intended for adoptive immunotherapy therefore remains insufficiently defined and warrants further investigation.

Recent studies have investigated the possibility of modulating tacrolimus activity by targeting its molecular mechanism. Genetic deletion of FKBP12 prevents the formation of the tacrolimus–FKBP12 complex, thereby restoring calcineurin activity. In this setting, Tconvs lacking FKBP12 retain normal activation, proliferation, and immune functionality despite tacrolimus exposure (20–23). However, this approach has not yet been evaluated in Tregs, and the impact of tacrolimus on this subset remains a subject of ongoing debate.

In this context, we hypothesize that FKBP12 depletion in Tregs may alleviate tacrolimus-mediated immunosuppression, thereby preserving their persistence in the presence of the drug. The objective of this study is to characterize the effects of tacrolimus on Treg proliferation, stability, and transcriptomic profile *ex vivo*, and to assess whether FKBP12 deletion can mitigate these effects.

## 2 Methods

### 2.1 PBMC isolation and Treg sorting

Human PBMCs from 20 healthy donors were isolated from leukoreduction system chambers (LRS) using density gradient centrifugation with Lymphoprep™ (STEMCELL Technologies) as previously described (44). CD25^+^ cells were enriched using magnetic beads (Miltenyi Biotec GmbH) following the manufacturer’s protocol. CD25^+^ and CD25^−^ cells were stained with a fixable viability dye and flow cytometry antibodies for CD4, CD127, and CD25 (**Supplementary Table 1**). Tregs (CD4^+^ CD127^−^ CD25^high^) were sorted from the CD25^+^ fraction (**Supplementary Figure 1A**), and Tconvs (CD4^+^) were isolated from the CD25^−^ fraction using a BD FACSAria III cell sorter. Sorted cells were plated in 96-well plates containing RPMI 1640 culture medium (Wisent Bioproducts) supplemented with 10% human serum AB (HS) (Sigma-Aldrich), 1% penicillin streptomycin (Gibco-Thermo Fisher Scientific), 1% GlutaMAX (Gibco-Thermo Fisher Scientific), 1 mM sodium pyruvate (Gibco-Thermo Fisher Scientific), and recombinant human IL-2 (SteriMax Inc.) at 500 IU/mL for Tregs. Cells were stimulated using a monoclonal antibody complex (tetramer) targeting CD3, CD28, and CD2 (STEMCELL Technologies) at 25 μL/mL. The culture medium was replaced every 48 hours, preserving the IL-2 concentration.

### 2.2 FKBP12 CRISPR-Cas9 depletion

After 5 days of culture, the ribonucleoprotein complex (RNP) was assembled by incubating 40 pmol of SpCas9 Nuclease Glycerol-Free per 36.6 pmol of synthetic sgRNA (5’-GGGCGCACCTTCCCCAAGCG-3’) (both from Integrated DNA Technologies) at 37 °C for 30 minutes. Cells were washed twice with RNase-free D-PBS (Multicell) and resuspended in Buffer R (Thermo Fisher Scientific) at a final concentration of up to 20 × 10^6^ cells/mL. Per electroporation, 9 μL of cell suspension was combined with 1 μL of RNP and 2 μL of Alt-R Cas9 Electroporation Enhancer at 10.8 μM in IDTE pH7.5 solution (both from Integrated DNA Technologie). Electroporation was performed using the Neon™ NxT Electroporation System 10-μL (Thermo Fisher Scientific) under the following conditions: 1400 V, 30 ms, single pulse. Immediately, cells were transferred into pre-warmed, antibiotic-free culture medium with IL-2 at 500 IU/mL and restimulated using the same antibody complex as initially. The culture medium was replaced every 48 hours, preserving the IL-2 concentration.

### 2.3 Tacrolimus treatment and cell quantification

Seven days after the deletion of FKBP12, which allows for protein breakdown, cells were counted and seeded in a complete growth medium containing 10 ng/mL tacrolimus (Sigma-Aldrich) or an equal volume of dimethyl sulfoxide (DMSO—Sigma-Aldrich) as a control. The cultures were exposed to different concentrations of IL-2 (100 IU/mL, 250 IU/mL, and 500 IU/mL) and restimulated using CD2/CD3/CD28 tetramers as initially. Every 48 hours, the culture medium was replaced with the appropriate concentration of IL-2 and tacrolimus or DMSO. After 7 days of treatment, cells were counted and processed for downstream analyses.

### 2.4 Nuclei isolation and nuclear NFAT1 staining

To isolate and stain nuclear NFAT1, we adapted a previously established protocol (24). Seven days after FKBP12 deletion, Tregs were cultured for 24 h in reduced IL-2 medium (100 IU/mL). Cells were then washed, resuspended at 1–2 × 10^6^ cells/mL in IL-2–free medium. Tacrolimus (10 ng/mL) or DMSO (control) was added, and cells were incubated for 60 min at 37 °C. Cells were then stimulated or not with IL-2 (final 100 IU/mL) and CD2/CD3/CD28 tetramers (final 12.5 µL/mL) for 60 min at 37 °C. Nuclei were isolated by incubation in ice-cold Buffer A, then B as previously described. Nuclei were then fixed and permeabilized using the Foxp3/Transcription Factor Staining Buffer Set, and intranuclear NFAT1 staining was performed for 60 min at 4 °C. After washing, nuclei were resuspended in cold FACS buffer (PBS, 10% FBS, 8 mM MgCl_2_). Buffer A and B compositions are provided in the **Supplementary Data** (**Supplementary Table 1**).

### 2.5 Flow cytometry

Extracellular staining was performed using antibody master mixes prepared according to experimental requirements, diluted in PBS containing 50% Brilliant Stain Buffer (BD Biosciences). Cells were incubated with the extracellular antibody mix for 30 minutes at 4 °C. Following extracellular staining, cells were fixed and permeabilized using the eBioscience™ Foxp3/Transcription Factor Staining Buffer Set (Thermo Fisher Scientific). Intracellular staining was then carried out by incubating cells with the antibody master mix in the permeabilization buffer for 45 minutes at RT. Samples were acquired using the Cytek^®^Aurora spectral flow cytometer (Cytek Biosciences), and data were analyzed with FlowJo software v10.10.0 (Becton, Dickinson & Company). A list of all antibodies and their respective clones is available in supplementary data (**Supplementary Table 2**).

### 2.6 Proliferation assay

Seven days after FKBP12 knockout, cells were counted and resuspended in PBS at 1–2 × 10^7^ cells/mL (≥0.5 mL), and a 20 µM cell-proliferation dye eFluor 450 (CPD eF450) (Thermo Fisher Scientific) working solution was prepared in PBS at RT. Equal volumes of cells and dye were mixed and incubated for 20 min at RT, then staining was quenched by adding cold RPMI with 10% FBS to a final volume of 14 mL and incubating for 5 min at 4 °C. Cells were centrifuged, the supernatant was discarded, and the pellet was washed once with complete medium without IL-2. After counting, Tregs were adjusted to 1 × 10^6^ cells/mL and 100 µL per well were seeded into 96-well round-bottom plates. Wells were then brought to 200 µL with medium to complete 10 ng/mL tacrolimus or an equivalent volume of DMSO, together with IL-2 (0, 100, 250, or 500 IU/mL). Cells were stimulated with the previously described CD2/CD3/CD28 tetramer mix at 5 µL/mL and cultured at 37 °C 4 days before analysis. Following culture, cells were stained extracellularly and samples were acquired by flow cytometry as described previously. Proliferation was quantified based on CPD dilution. The division peaks were used to calculate the proliferation index using the Proliferation Modeling tool in FlowJo v10.10.0 (Becton, Dickinson & Company). Proliferation was calculated as 100-[(proliferation index of the sample/proliferation index of untreated control Tregs at 500 IU/mL) × 100]. A complete list of antibodies used is provided in the **Supplementary Material** (**Supplementary Table 1**).

### 2.7 Suppression assay

Allogeneic PBMCs were labeled with CPD ef450, and Tregs with CPD ef670 CDP (ThermoFisher Scientific). A total of 1 × 10^5^ PBMCs per well were plated in a 96−well plate and stimulated with CD3/CD28 Dynabeads (ThermoFisher Scientific) at a 1:16 cell−to−bead ratio. Labeled Tregs were added at the specified ratios (1:1, 1:2.5, 1:5, 1:10, 1:20 and 1:40) and cultured at 37 °C. After 4 days, cells were stained for extracellular markers (CD3, CD4, CD8) using flow cytometry. Division peaks of CD4^+^ and CD8^+^ cells were used to calculate the division index using the Proliferation Modeling tool in FlowJo v10.10.0 software (Becton, Dickinson & Company). Suppression was calculated as 100-[(Division index with Tregs/Division index without Tregs *100)].

### 2.8 Pro−inflammatory cytokine challenge

Seven days after FKBP12 knockout, cells were counted and plated in a 96−well plate. Cells were restimulated with CD2/CD3/CD28 tetramers as initially. Cultures were maintained in the presence of IL−2 (500 IU/mL), TNF−α (20 ng/mL), IL−1β (20 ng/mL), IL−6 (20 ng/mL), and IFN−γ (20 ng/mL) for 7 days, with medium replaced every 48 hours. Flow cytometry staining was then performed as previously described.

### 2.9 Indel quantification

A subset of cultured cells (≥1 × 10^5^ cells) was preserved for genomic DNA extraction using the Monarch Spin gDNA Extraction Kit (New England Biolabs), following the manufacturer’s guidelines. DNA concentration and quality were evaluated using the spectrophotometer Infinite^®^ M1000 PRO (Tecan Group Ltd.). PCR amplification of the sgRNA-targeted genomic region was performed using OneTaq Hot Start 2X Master Mix with GC Buffer (New England Biolabs). PCR reactions were run on the MiniAmp™ Thermal Cycler (Thermo Fisher Scientific). Amplification success was confirmed by agarose gel electrophoresis, and PCR products were cleaned using the Monarch^®^ PCR & DNA Cleanup Kit (New England Biolabs). Samples were submitted for Sanger sequencing using the Nextera Kit (Illumina) on a 3730 ABI Genetic Analyzer (Thermo Fisher Scientific). Sequencing data (FASTA format) were compared, and knockout efficiency was quantified using ICE (Inference of CRISPR Edits) online software (Synthego). Furthermore, the Guide Verification tool version 1.3 (Synthego) was used to perform an *in silico* off-target analysis of the used sgRNA. The predicted off-target sites are summarized in supplementary data (**Supplementary Table 3**).

### 2.10 RNA-seq

Cells were lysed directly in TRIzol Reagent (Thermo Fisher Scientific) and stored at −80 °C. After thawing, phase separation was initiated by adding chloroform. Samples were centrifuged (12, 000 x g, 4 °C) to isolate the aqueous phase. The upper aqueous layer was collected, and an equal volume of 100% ethanol (Greenfield Global) was added. RNA purification was then carried out using the Monarch^®^Total RNA Miniprep Kit (New England Biolabs), beginning at the “Capture Total RNA” step, in accordance with the manufacturer’s instructions. RNA was quantified using Qubit™ (Thermo Fisher Scientific) and quality was assessed with a 2100 Bioanalyzer (Agilent Technologies). Transcriptome libraries were generated using the KAPA RNA HyperPrep kit (Roche) and a poly-A selection (Thermo Fisher Scientific). Sequencing was performed on the Illumina NextSeq2000, obtaining around 30x10^6^ single-end 100bp reads per sample.

### 2.11 Bio-informatic analysis

The analysis of raw gene-level count matrices was performed using R version 4.4.0 and edgeR version 4.4.2. A global and most robust approach was taken with the implementation of quasi-likelihood (QL) GLM modelling, while single factor comparisons were carried out using the classical exact test. The selection of significant differentially expressed genes was made using the following cutoffs: P-value < 0.05 and |logFC| ≥ 1.

### 2.12 Statistical analysis

All statistical analyses were done using Prism version 10.6.1. To compare three or more conditions, a one-way ANOVA test was done in a pairing mixed-effects analysis, with the Geisser-Greenhouse correction. For matched repeated-measures data across two within-subject factors (Condition × Ratio), data were analyzed using a two-way repeated-measures ANOVA, with Donor treated as the matched variable and the Geisser–Greenhouse correction applied. (****P ≤ 0.0001, ***P ≤ 0.001, **P ≤ 0.01, *P ≤ 0.05).

## 3 Results

### 3.1 Tacrolimus inhibits human Treg proliferation in an FKBP12-dependent manner

**Figure 1:**
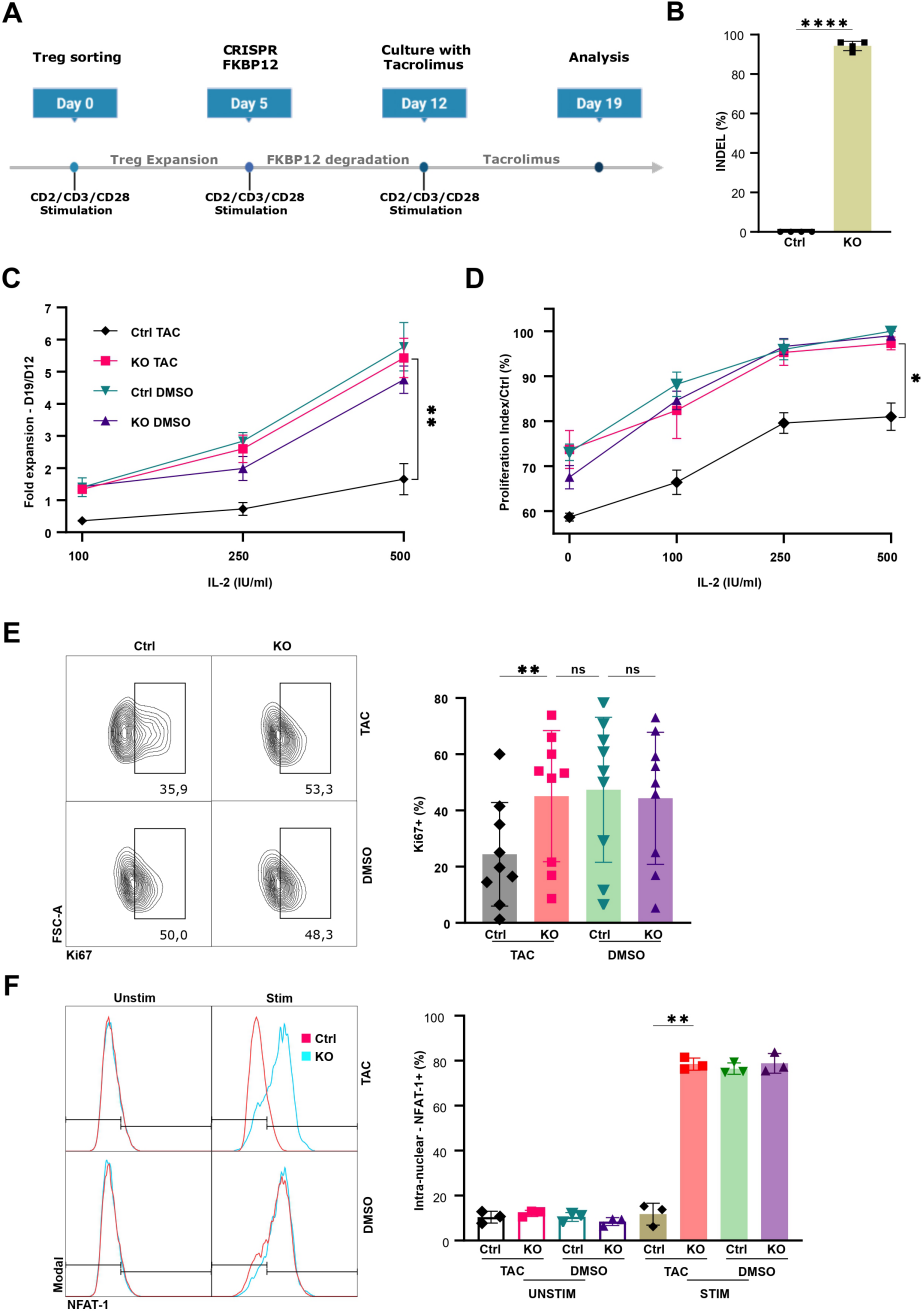
Genetic disruption of FKBP12 uncouples Treg proliferation from tacrolimus-mediated inhibition. **(A)** Experimental timeline. Isolated human Tregs were expanded, edited with CRISPR–Cas9 at day 5 to target FKBP12, exposed to tacrolimus from day 12 to day 19, and analyzed. **(B)** Editing efficiency of the FKBP12 locus. The percentage of the edited sample with non-wild type sequence (INDEL%) was determined by Inference of CRISPR Edits (ICE) analysis. n=4 from 2 independent experiments. **(C)** Fold expansion (D19/D12) across IL-2 doses (100–500 IU/mL) under tacrolimus (10 ng/mL) or DMSO. n=3–7 from 4 independent experiments **(D)** Proliferation index using a cell proliferation dye assay measured over 4 days starting at day 12 and normalized to control using Flowjo proliferation tool. n=3 from 2 independent experiments, **(E)** Ki-67 expression analysis at 500 IU/mL IL-2: representative contour plots (left) and summary data (right). n= 9 from 5 independent experiments **(F)** NFAT1 nuclear translocation analysis after 1 hour of CD2/CD3/CD28 activation in the presence of 100 IU/mL IL−2 and either tacrolimus (10 ng/mL) or DMSO. Representative histograms (left) and summary data (right). n = 3 from two independent experiments. ****P ≤ 0.0001, **P ≤ 0.01, *P ≤ 0.05, ns = not significant.

To investigate the effect of tacrolimus on human Tregs and its underlying mechanisms, Tregs were isolated from the PBMCs obtained from leukoreduction system chambers of healthy donors (**Supplementary Figure 1A**). Cells were first expanded *in vitro* for 5 days before the FKBP12 gene was depleted by CRISPR-Cas9. The edited cells were then maintained under standard culture conditions for an additional week to allow the degradation of residual FKBP12 protein. Subsequently, Tregs were restimulated and cultured for 7 days in the presence of a therapeutically relevant concentration of tacrolimus (10 ng/mL) together with varying concentrations of IL-2 (100, 250 or 500 IU/mL) (**Figure 1A**). Cells were paired across all four conditions to control for donor-specific variability. The efficiency of FKBP12 depletion was evaluated at the genomic level by sequencing PCR amplicons, followed by ICE analysis comparing knockout (KO) and control samples (Ctrl). This analysis revealed a high degree of FKBP12 gene disruption across the cell population, indicating efficient editing and supporting the robustness of the downstream functional readouts (**Figure 1B**; **Supplementary Figure 2**).

Tacrolimus treatment led to a marked reduction in Treg proliferation, as determined by cell counts, whereas FKBP12-KO Tregs were largely refractory to this effect. This protective effect of FKBP12 depletion was consistently observed across all IL-2 concentrations tested (**Figure 1C**). FKBP12 depletion did not significantly alter Treg proliferation under standard culture conditions (**Figure 1C**). In parallel, labeling with a cell proliferation dye (CPD) across all conditions allowed the quantification of division numbers and calculation of the proliferation index. Tacrolimus markedly reduced the proliferation index in control Tregs, whereas FKBP12-KO Tregs maintained proliferation profiles similar to untreated cells (**Figure 1D**). To further characterize proliferation dynamics, we assessed the expression of the proliferation-associated nuclear factor Ki-67. Tacrolimus induced a significant decrease in Ki-67 expression in control Tregs, while FKBP12-KO Tregs retained Ki-67 levels comparable to untreated cells (**Figure 1E**).

Given that tacrolimus inhibits NFAT nuclear translocation, we next assessed NFAT localization in Tregs. In control Tregs, tacrolimus almost completely abrogated the increase in nuclear NFAT1 normally induced by CD2/CD3/CD28 and IL-2 stimulation, whereas FKBP12-KO Tregs maintained a strong NFAT1 nuclear signal despite tacrolimus exposure, comparable to untreated stimulated cells. These data indicate that tacrolimus impairs NFAT activation in human Tregs in an FKBP12-dependent manner (**Figure 1F**). Together, these findings demonstrate that the antiproliferative effect of tacrolimus on human Tregs is critically and specifically dependent on FKBP12.

### 3.2 FKBP12 knockout does not compromise human Treg phenotype

**Figure 2:**
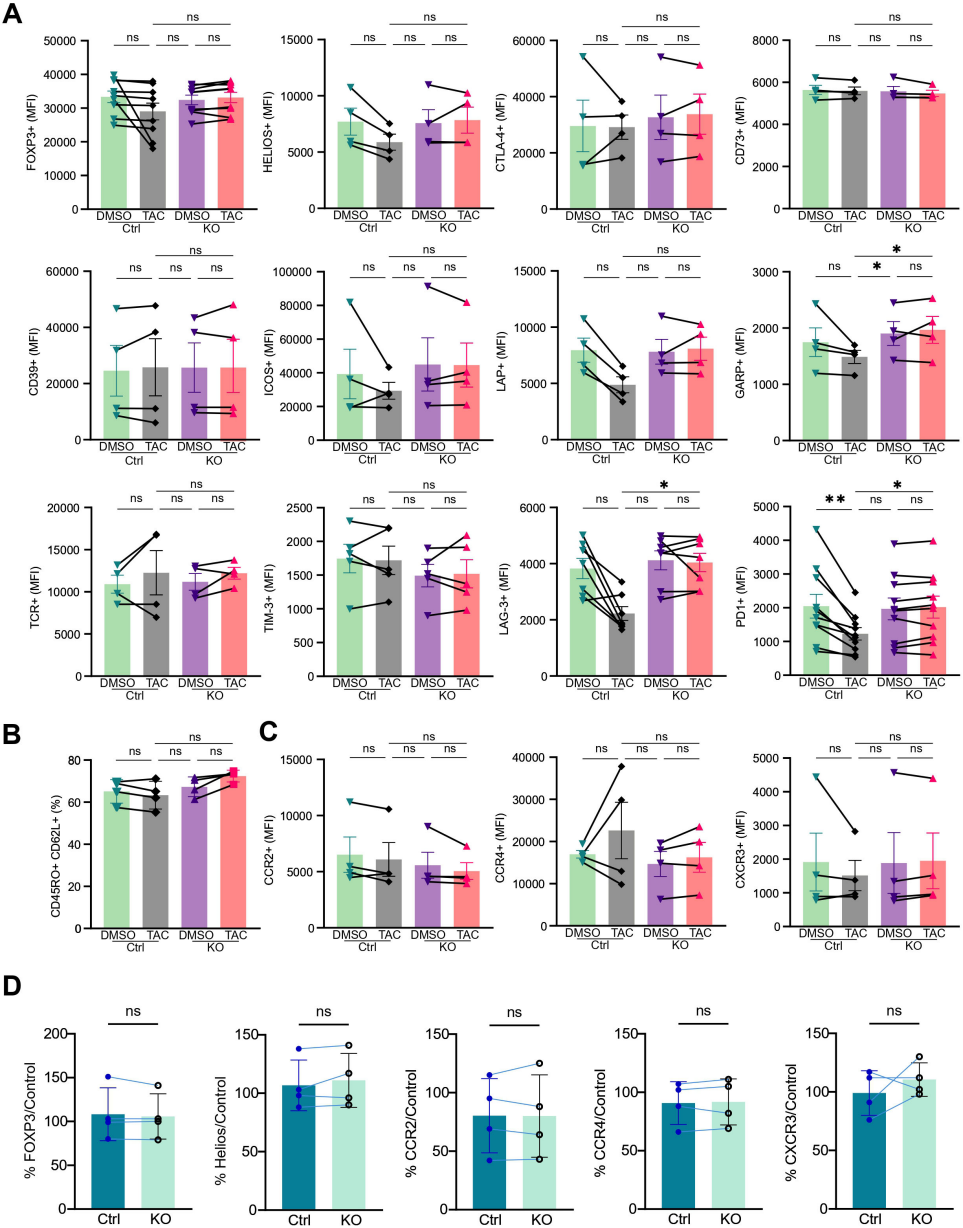
FKBP12 knockout preserves Treg phenotype. Flow-cytometric phenotyping at day 19 at 500 IU/mL of IL-2. **(A)** Comparison of median fluorescence intensity (MFI) of FOXP3, HELIOS, CTLA-4, CD73, CD39, ICOS, LAP, GARP, TCRαβ, TIM-3, LAG-3, and PD-1 between DMSO and tacrolimus (tac) conditions in control (Ctrl) and FKBP12 KO Tregs. **(B)** Frequency of central memory Tregs (CD45RO^+^CD62L^+^). *n* = 4–9 from 2–5 independent experiments. **(C)** MFI of CCR2-, CCR4-, and CXCR3-positive cells. *n* = 4. Each point represents an individual donor (paired across all four conditions), and lines connect matched DMSO and tacrolimus within CTRL or KO donors for visualization purposes only. **(D)** Treg phenotype under pro-inflammatory challenge. % expression of FOXP3, HELIOS, CCR2, CCR3 and CXCR3 under inflammatory conditions, with or without FKBP12 depletion, compared to controls. n=4. Bars represent the mean ± SEM. **P ≤ 0.01, *P ≤ 0.05, ns = not significant.

An important safety consideration for any genetically engineered Treg product is whether the manipulation perturbs lineage stability, differentiation, expression of co-inhibitory receptors associated with suppressive function or exhaustion and homing ability. We therefore comprehensively profiled FKBP12-deficient and control Tregs by flow cytometry and bulk RNA-seq after culture in 500 IU/mL IL-2, with or without tacrolimus. At the protein level, FKBP12 deletion had no significant impact on FOXP3, HELIOS, CTLA-4, CD73, CD39, ICOS, LAP, TCR or TIM-3 expression (**Figure 2A**; **Supplementary Figures 1B**, **3**–**5**). We only observed a decrease in GARP, LAG-3 and PD-1 expression in control Tregs in the presence of tacrolimus, possibly reflecting a decrease in cell activation (**Figure 2A**; **Supplementary Figure 5**). Likewise, tacrolimus itself did not markedly alter these markers relative to untreated cells. By day 19 of culture, the majority of cells in all conditions had transitioned to a central memory CD45RO^+^CD62L^+^ phenotype, again with no meaningful differences between FKBP12-KO and control Tregs or between tacrolimus-treated and untreated samples (**Figure 2B**). We also evaluated the surface expression of key chemokine receptors implicated in Treg migration to the graft, i.e., CCR2, CCR4 and CXCR3 (25) and found no significant differences between control and FKBP12-KO Tregs, either in the presence or absence of tacrolimus (**Figure 2C**), or after an inflammatory challenge (**Figure 2D**). In addition, transcriptomic analysis confirmed comparable mRNA expression levels of *CCR2*, *CCR4* and *CXCR3* between conditions (**Supplementary Figure 6**).

To evaluate FKBP12-KO Treg stability, we also expanded them for 7 days under proinflammatory conditions (IL-2 together with TNF-α, IL-1β, IL-6, and IFN-γ). Under these conditions, we did not observe any reduction in FOXP3, HELIOS expression between control and FKBP12-KO Tregs (**Figure 2D**; **Supplementary Figure 1C**). These findings indicate that FKBP12 deletion does not measurably destabilize the Treg phenotype or skew maturation state under strong IL-2 stimulation, and that tacrolimus, in this context, primarily constrains proliferation rather than driving overt phenotypic reprogramming.

### 3.3 FKBP12 knockout does not compromise human Treg transcriptome or *in vitro* suppressive function

**Figure 3:**
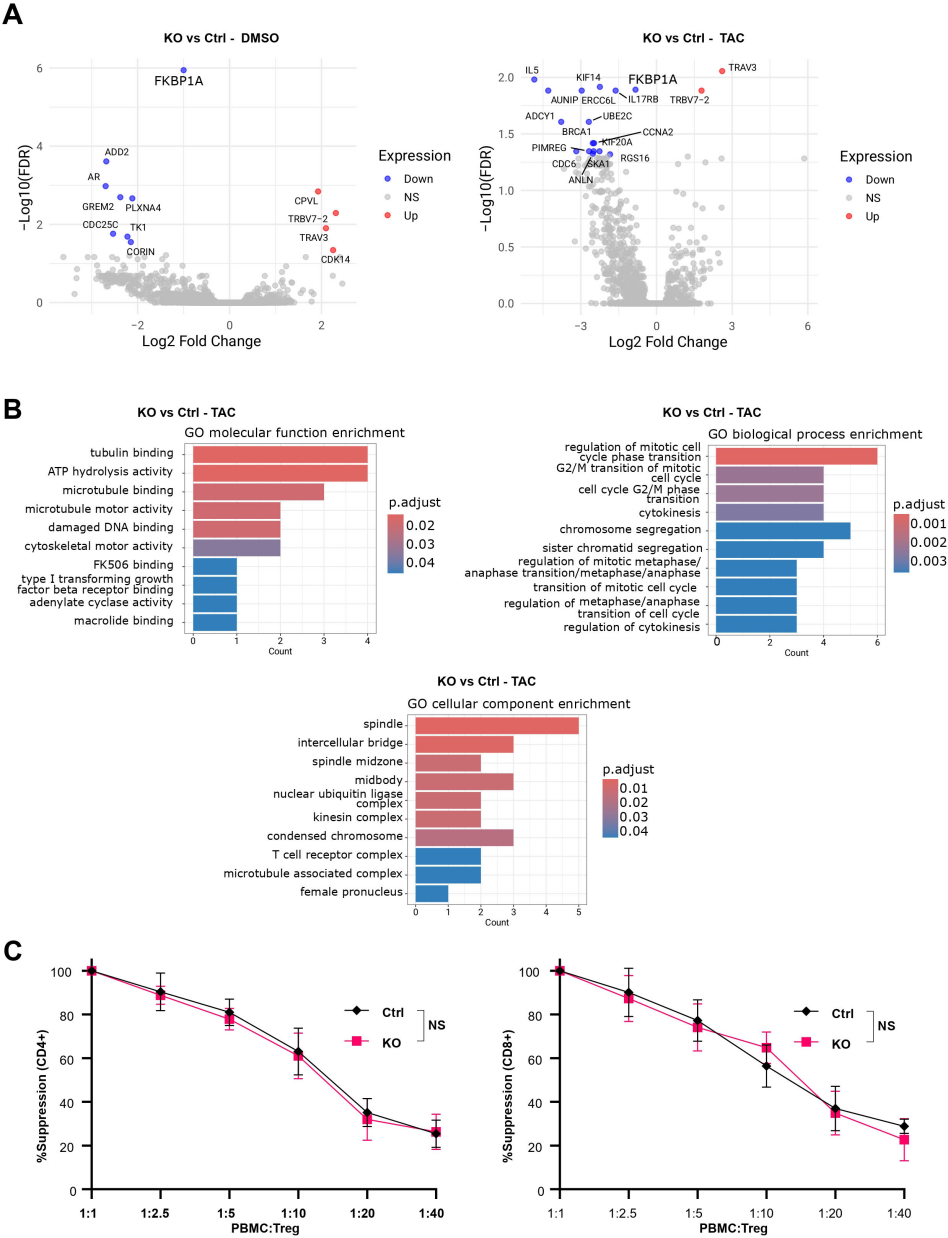
FKBP12 knockout causes only minimal transcriptomic remodeling and preserves Treg suppression *in vitro.***(A)** Volcano plots showing differentially expressed genes between KO vs Ctrl in DMSO (left) or Tacrolimus (right). **(B)** GO enrichment for KO vs Ctrl under tacrolimus (bar length = gene count; shade = p.adjust). **(C)** % Suppression of CD4^+^ (left) and CD8^+^ (right) Tconv proliferation by FKBP12-KO and control Tregs across Treg:effector ratios. n=4 from 2 independent experiments. NS = not significant.

To extend our analysis to the transcriptional level, we profiled the complete transcriptome of Tregs across all experimental conditions. A principal component analysis (PCA) did not reveal any clear segregation of samples based on FKBP12 status (**Supplementary Figure 7A**), indicating broadly similar global expression profiles. As expected, expression of *FKBP1A*, gene encoding FKBP12, was markedly reduced in KO cells compared with controls (**Supplementary Figure 7B**). Aside from this gene, only a very limited number of genes were differentially expressed between KO and control cells (**Figure 3A**). The biological coefficient of variation (BCV) was determined to be 0.26, which fall within the expected range for bulk RNA-seq of human cells and is consistent with moderate biological variability rather than excessive dispersion that could limit sensitivity for differential expression (26). Consistent with this, global gene expression profiles showed high concordance both within and between experimental groups (**Supplementary Figure 8**). Gene set and ontology analyses of tacrolimus-treated Tregs comparing KO versus control cells demonstrated that, within the cellular component, molecular function, and biological process categories, the most enriched pathways were predominantly related to cell cycle and proliferation (**Figure 3B**). Together, these transcriptomic analyses indicate that FKBP12 deletion does not induce broad changes in Treg identity.

We therefore next examined whether preservation of the transcriptional program translated into maintained Treg suppressive function. Suppressive capacity, evaluated using a 4-day *in vitro* suppression assay, was comparable between FKBP12-KO and control Tregs, indicating that enhanced proliferative responses did not compromise Treg function (**Figure 3C**).

## 4 Discussion

Compatibility between tacrolimus and optimal human Treg biology remained an open question, as the literature reported mixed outcomes (11, 12). Our study addressed this question using a mechanistic approach based on CRISPR–Cas9 deletion of the FKBP12 gene in *ex vivo* expanded human Tregs. We showed that a therapeutically relevant dose of tacrolimus exerts a significant, FKBP12-dependent antiproliferative effect on human Tregs, even in the presence of high-dose IL-2, while leaving their core lineage phenotype, global transcriptomic profile and suppressive function basically intact. These findings are consistent with the canonical mechanism of tacrolimus, whereby FKBP12–tacrolimus complexes inhibit calcineurin, preventing NFAT dephosphorylation and thereby blocking transcriptional programs required for T-cell proliferation (4). FKBP12 KO-Tregs normally proliferate under tacrolimus and remain phenotypically indistinguishable from untreated control cells, indicating that genetic disruption of the tacrolimus–FKBP12 axis decouples Treg proliferation from calcineurin-inhibitor signaling and preserves Treg identity.

Our data align with prior *in vitro* work showing that tacrolimus suppresses the proliferation of human Tregs, while exerting a limited effect on their phenotype. Other teams reported that cyclosporine A and tacrolimus reduced Treg proliferation, but only cyclosporine, at low doses, compromised Treg function and cytokine profile (27). This supports the view that Treg proliferation is highly sensitive to calcineurin pathway interference, even when core lineage stability is maintained. Scottà et al. similarly found that several standard immunosuppressants, including tacrolimus, significantly impaired the proliferative capacity and *in vivo* efficacy of *ex vivo* expanded human Tregs intended for cell therapy (2). The main exception being the mTOR inhibitor sirolimus, which can more selectively spare or even expand Tregs (11, 12, 28). However, it is not routinely used because of its numerous side effects.

The NFAT1 nuclear translocation data provides direct mechanistic support for FKBP12-dependent tacrolimus inhibition in human Tregs. Consistent with prior studies (24, 29, 30), CD2/CD3/CD28 plus IL-2 stimulation induced a robust increase in nuclear NFAT1 in control Tregs, which was markedly suppressed by tacrolimus. In contrast, FKBP12-KO Tregs maintained NFAT1 nuclear localization despite tacrolimus exposure, indicating restoration of calcineurin-NFAT signaling. The stimulation strategy used delivered integrated TCR and co-stimulatory signals that approximate antigen-presenting cell–mediated activation and is widely used for clinical-grade T cell expansion (31). Given the threshold-dependent nature of NFAT activation downstream of TCR signaling, these findings suggest that FKBP12-dependent NFAT blockade may similarly constrain antigen-specific or engineered Treg products exposed to tacrolimus.

By genetically ablating FKBP12, we confirmed that the antiproliferative effect of tacrolimus on human Tregs is strictly dependent on this chaperone and, by extension, on calcineurin–NFAT inhibition. This is in line with earlier work showing that FKBP12 is the dominant FK506-binding protein responsible for T-cell inhibition by tacrolimus in Tconvs (4). Our study extends this paradigm to human Tregs and demonstrates that FKBP12 deletion effectively renders Tregs “blind” to tacrolimus with respect to proliferation.

An unexpected observation is that supraphysiologic IL-2 concentrations failed to overcome the antiproliferative effect of tacrolimus in human Tregs. Even at 500 IU/mL IL-2, tacrolimus markedly reduced cell expansion, Ki-67 expression, and proliferation index in control Tregs, whereas FKBP12-KO Tregs proliferated normally. This contrasts with reports in effector T cells where strong IL-2 receptor (CD25) signaling can partially compensate for calcineurin blockade. For example, IL-2 supplementation has been shown to restore certain functional aspects of CNI-treated effector T cells (32–34) and to correct CNI-treated affecting mince-Treg dysfunction in some experimental models (13, 17).

Mechanistically, Treg biology is uniquely dependent on IL-2, but IL-2 signaling alone is not sufficient to drive optimal expansion in the absence of TCR–NFAT input. IL-2–STAT5 pathways promote Treg survival, FOXP3 expression, and suppressive function (35, 36), whereas NFAT cooperates with FOXP3 at key regulatory elements to sustain the Treg transcriptional program (6). Our data suggest that in the context of potent calcineurin inhibition, even high-dose IL-2 cannot fully substitute for NFAT-dependent signals required for Treg cell cycle entry and progression. This may reflect a greater reliance of Tregs than Tconvs on integrated TCR–NFAT and IL-2–STAT5 signaling for proliferation, such that inhibition of calcineurin imposes a non-redundant bottleneck that IL-2 cannot bypass. Our findings therefore refine the emerging view that simply escalating IL-2 dosing is unlikely to correct CNI-induced proliferation defects in Tregs, particularly when tacrolimus levels are in the therapeutic range. This has important implications for clinical protocols that combine IL-2-based therapies with tacrolimus in Treg supportive regimens.

Our work builds directly on recent efforts to engineer tacrolimus-resistant cell therapy with Tconvs (20, 21, 37). We extended this concept to human Tregs and showed that FKBP12 deletion confers tacrolimus resistance at the level of proliferation without detectable disruption of lineage markers, phenotype, or global gene expression. This is consistent with the broader Treg literature, where FOXP3 expression and lineage stability are maintained by a robust transcriptional and epigenetic network that is relatively resilient to individual signaling perturbations, particularly in the presence of ample IL-2 (6, 36). Our findings suggest that FKBP12 editing is unlikely to compromise Tregs’ favorable attributes, at least in the short term and under the culture conditions tested. In combination with the prior effector T-cell studies (20, 21, 37), this supports FKBP12 editing as a generalizable strategy to insulate defined T-cell subsets from CNI therapy. From a translational perspective, this could enable the co-administration of engineered Treg products alongside tacrolimus, supporting immunosuppression minimization strategies and potential progression toward tacrolimus monotherapy, as explored in the ONE (38), the TWO (39, 40) and the ongoing STEADFAST trials (38, 41). Our data add to accumulating evidence that tacrolimus, at clinically relevant levels, can impair the proliferative capacity of human Tregs even in IL-2–rich conditions. In the setting of adoptive Treg transfer, this may limit *in vivo* expansion, persistence, and tissue accumulation of infused cells, thereby constraining therapeutic efficacy.

At the same time, FKBP12 has additional roles beyond calcineurin regulation, including interactions with ryanodine receptors and TGF-β signaling components and epigenetic changes (42, 43), raising theoretical concerns about the long-term consequences of its deletion. While our transcriptomic data did not reveal obvious perturbations in major signaling pathways, more detailed functional assessment, particularly of calcium signaling, metabolic fitness, and *in vivo* persistence will be needed before broad clinical implementation.

CRISPR–Cas9 editing inevitably raises concerns about off-target cleavage. However, the sgRNA used in this study was carefully selected to ensure high specificity and reduce the likelihood of unintended cleavage (20). This sgRNA has previously been used under GMP-like conditions to generate tacrolimus-resistant antiviral and SARS-CoV-2–specific T-cell products; in these studies, FKBP12-KO cells maintained normal expansion kinetics, TCR repertoire diversity, and effector function (21). Consistently, our own *in silico* evaluation predicted a restricted set of candidate off-target loci, and FKBP12-KO Tregs in this study retained a typical phenotype, sustained FOXP3 expression, and a transcriptomic profile closely matching control Tregs.

Several limitations of this study should be acknowledged. First, although FKBP12-deficient Tregs were shown to retain phenotypic stability, transcriptomic identity, and suppressive function *in vitro*, the long-term consequences and epigenetic impact of FKBP12 deletion were not assessed. Potential effects on lineage stability, fitness, or function following prolonged expansion or *in vivo* persistence remain to be determined. Second, all experiments were performed using Tregs isolated from healthy donors. While this represents a standard and necessary first step for mechanistic and manufacturing studies, Tregs derived from patients with end-stage kidney disease or on dialysis, who would constitute the target population for future clinical translation, may display distinct features. Nevertheless, there is no *a priori* reason to expect that disruption of the FKBP12–calcineurin interaction would differentially affect the fundamental proliferative response to tacrolimus in patient-derived Tregs.

In addition, transcriptomic analyses were conducted with a limited number of biological replicates (n = 3 per condition), which may reduce sensitivity for detecting subtle differential gene expression. To mitigate this limitation, we applied multiple complementary analytical approaches, including assessment of biological variability and global expression concordance, which collectively support the conclusion that FKBP12 deletion does not induce major transcriptional reprogramming. Finally, the Tregs used in this study were polyclonal rather than antigen specific. While this approach is well suited for evaluating core mechanisms of tacrolimus resistance and Treg expansion, future studies will be required to determine how FKBP12 editing performs in antigen-specific or engineered Treg products intended for targeted immunoregulation.

In summary, we demonstrated that tacrolimus exerts a potent, FKBP12-dependent antiproliferative effect on human Tregs that persists even at high IL-2 concentrations, while leaving Treg phenotype, maturation markers, global gene expression and suppressive ability largely preserved. FKBP12 deletion renders Tregs resistant to tacrolimus at the level of proliferation without overtly compromising lineage identity, thereby extending FKBP12-editing strategies previously validated in effector T cells. Together, these findings support FKBP12-edited Tregs as a promising platform for next-generation adoptive Treg immunotherapy.

## Data Availability

The datasets presented in this study can be found in online repositories. The names of the repository/repositories and accession number(s) can be found below: https://www.ncbi.nlm.nih.gov/sra/PRJNA1365938.
