# Supplementary material for: Engineering human Tregs to resist tacrolimus via FKBP12 gene editing
Source: Front Immunol. 2026 Apr 17;17:1756624. doi: 10.3389/fimmu.2026.1756624 (PMC13133020; doi:10.3389/fimmu.2026.1756624)
Supplement: Supplementary file 1 [file DataSheet1.pdf]

Supplementary Material

1.1 Supplementary Figures

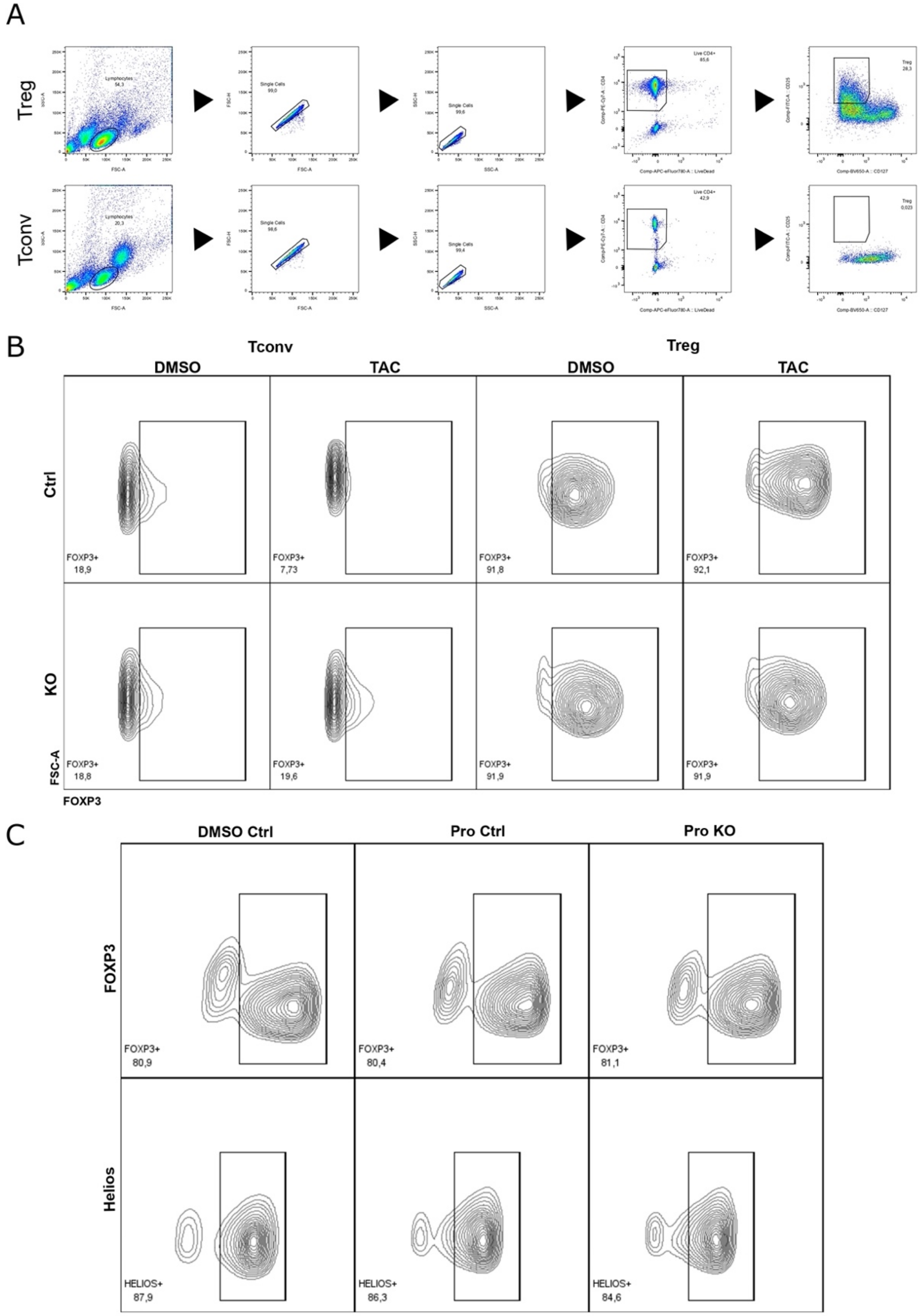

Supplementary Figure 1. Human Treg sorting strategy and representative cytometry plots.
(**A**) Gating strategy to isolate Tregs and Tconvs from LRS-chamber PBMCs. Sequence: 1) Lymphocytes (FSC-A/SSC-A) 2) doublet exclusion (FSC-H vs FSC-A; SSC-H vs SSC-A) 3) viable cells (FVD$^-$ CD4$^+$) 4) Tregs (CD25^{high} CD127$^{low/–}$) **(B)** Representative flow cytometry plots at day 19 with IL-2 under tacrolimus (TAC) or DMSO, in Ctrl and KO conditions in both Treg and conventional CD4$^+$ T cells. FOXP3 frequencies remain high in Tregs. **(C)** Representative flow cytometry plots at day 19 with IL-2 with either DMSO or in pro-inflammatory condition (Pro) in Ctrl and KO conditions. FOXP3 and Helios frequencies remain similar despite pro-inflammatory cytokines.

A

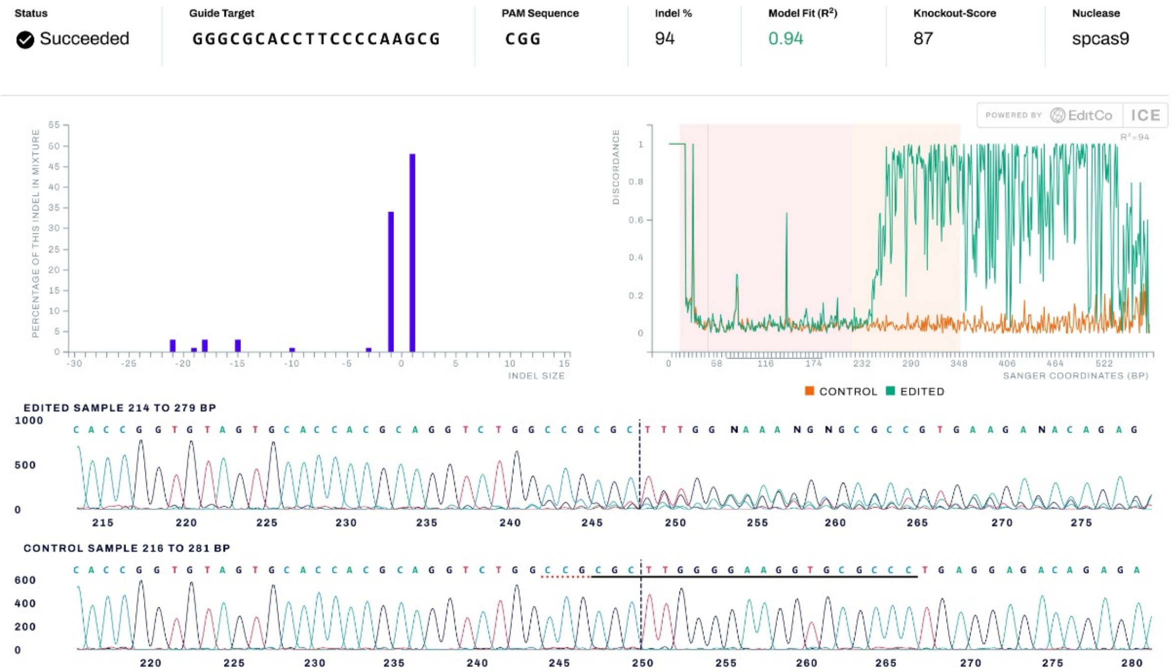

Supplementary Figure 2. Representative ICE analysis confirming efficient FKBP1A editing.

(A) ICE report (Sanger deconvolution) for a representative donor edited with SpCas9 at FKBP1A. Summary metrics: guide target sequence, PAM = CGG, Indel = 94%, KO-score = 87 (predicted fraction of frameshift alleles), and Model fit (R^2) = 0.94. The indel-size histogram indicates a distribution dominated by short events around the cut site, while the discordance plot shows trace divergence starting at the PAM/cleavage region. Bottom panels display edited vs control Sanger chromatograms with mixed peaks downstream of the cut, consistent with a polyclonal edited population. Collectively, the data support a near-quantitative edit compatible with functional FKBP12 knockout.

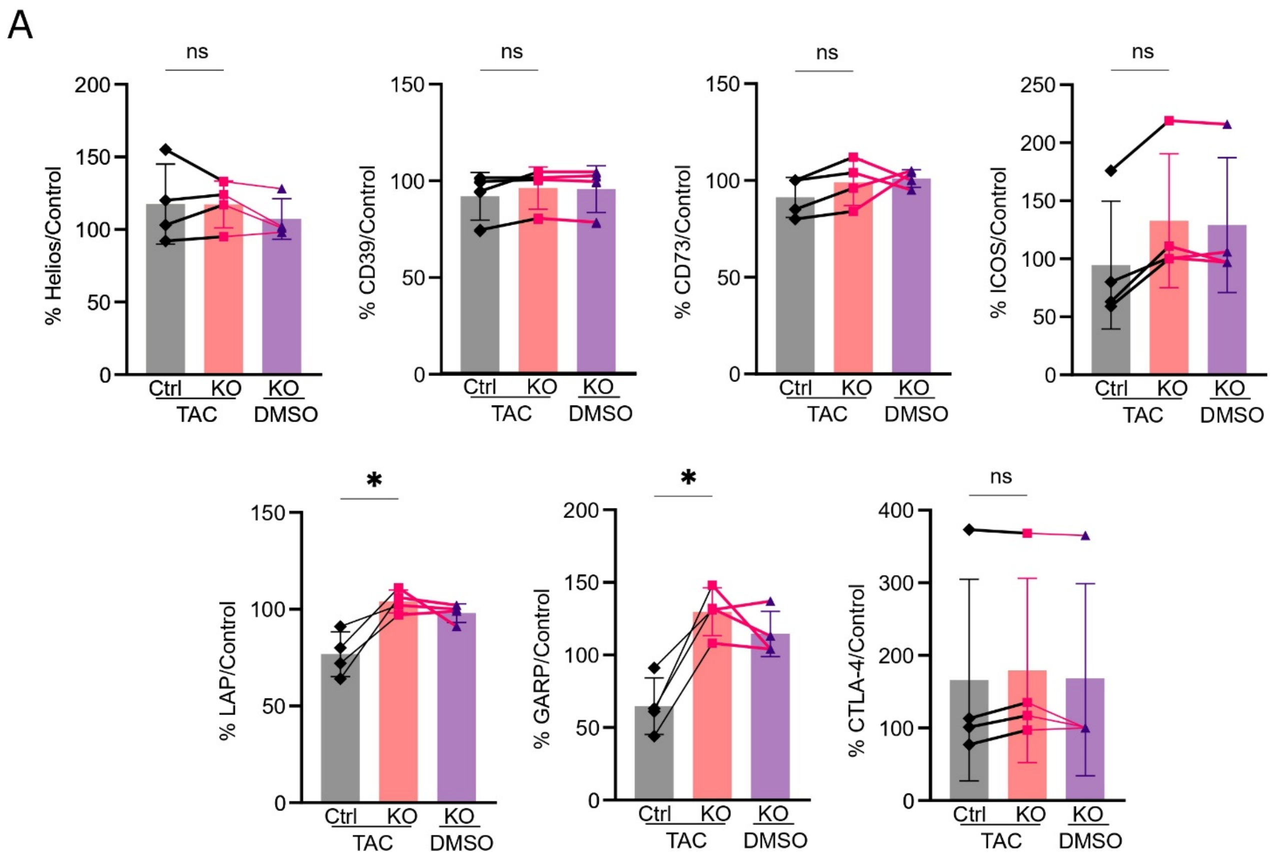

Supplementary Figure 3. Normalized expression of Treg-associated protein per donor.

Due to inter-donor variability in baseline expression levels, the expression of Treg-associated proteins was normalized to the control condition for each individual donor to better highlight the impact of FKBP12 depletion. Data are presented as percent change relative to paired control Tregs from the same donor. Expression of Helios, CTLA-4, CD39, CD73, ICOS, LAP, and GARP was assessed by flow cytometry.

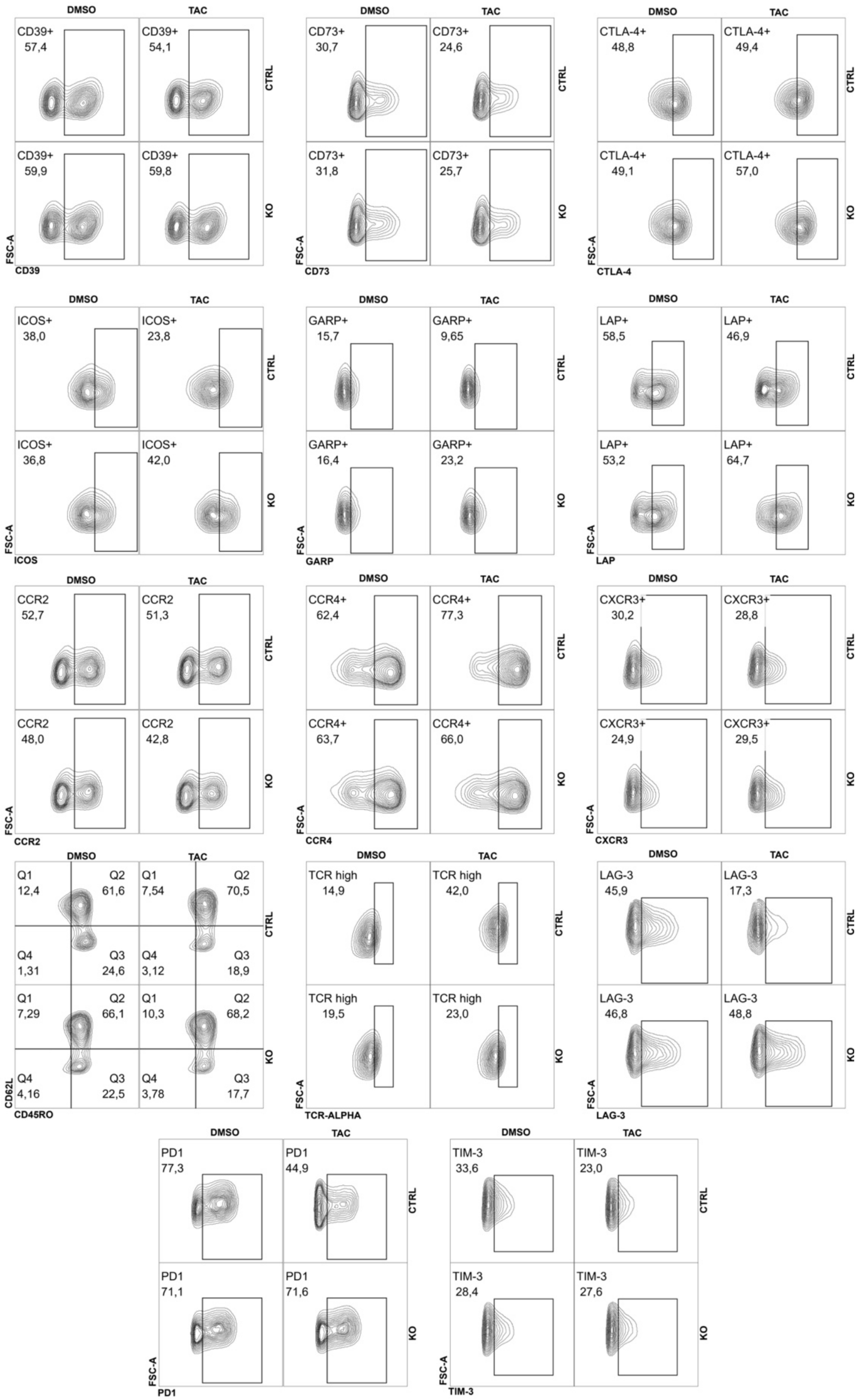

Supplementary Figure 4. Representative cytometry plots.

Representative flow cytometry plots at day 19 with IL-2 under tacrolimus (TAC) or DMSO, in Ctrl and KO conditions.

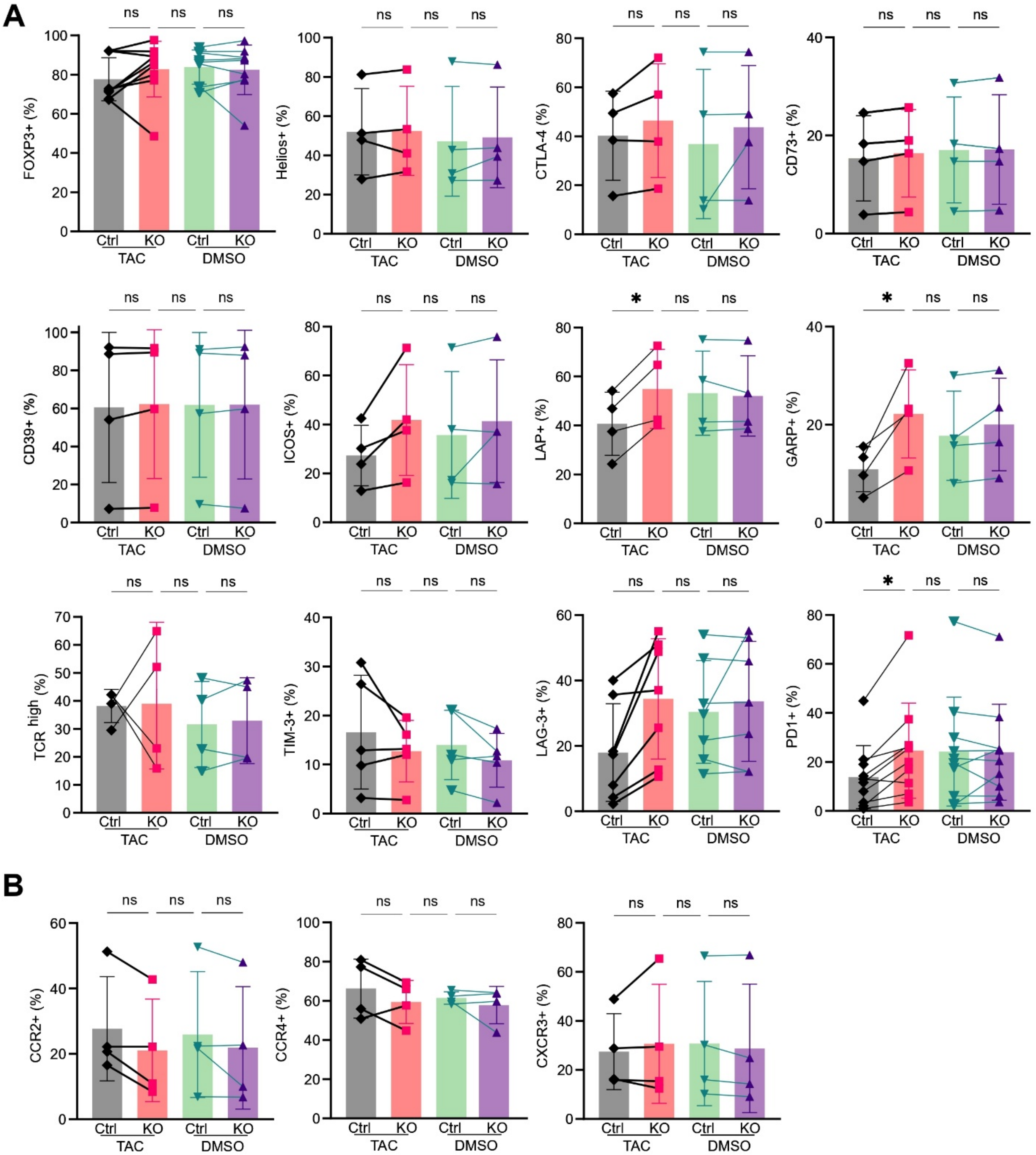

Supplementary Figure 5. Flow-cytometry phenotyping (%) at day 19

(A) Flow-cytometry phenotyping at day 19 at 500 IU/mL IL-2. **(A)** % positive FOXP3, HELIOS, CTLA-4, CD73, CD39, ICOS, LAP, GARP, TCRαβ^{high}, TIM-3, LAG-3, PD-1. n= 4-9 from 2-5 independent experiments. (B) % positive CCR2, CCR4 and CXCR3 n=4.

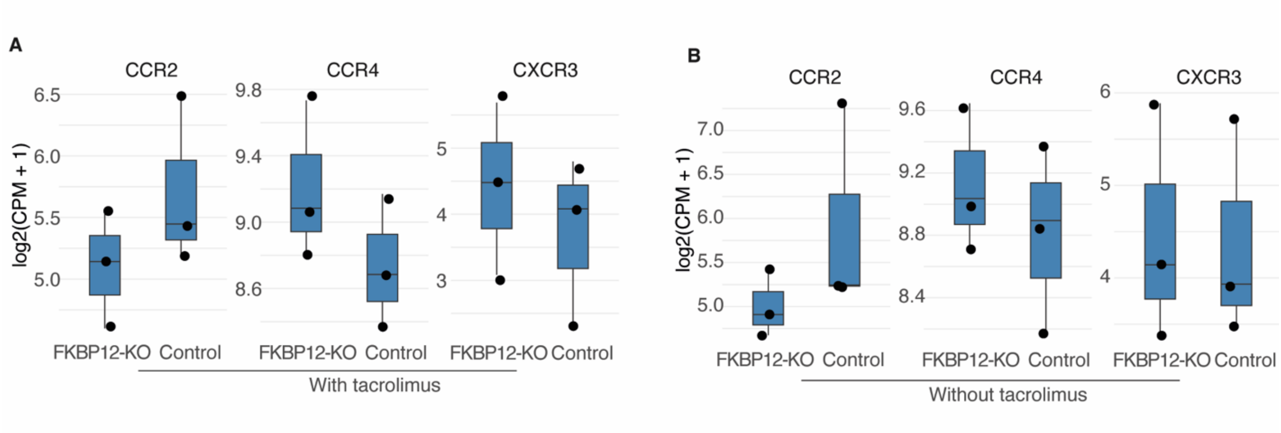

Supplementary Figure 6. Expression of chemokine receptor transcripts in FKBP12-KO and control Tregs.

RNA-seq–derived expression levels (log2 CPM) of *CCR2*, *CCR4*, and *CXCR3* in FKBP12-KO (KO) and control human Tregs cultured in the presence of tacrolimus (A) or vehicle control (B). Each dot represents an individual biological replicate (n = 3 per condition). Box plots indicate the median (center line), interquartile range (box), and range (whiskers). No statistically significant differences in chemokine receptor transcript levels were observed between KO and control Tregs under either condition.

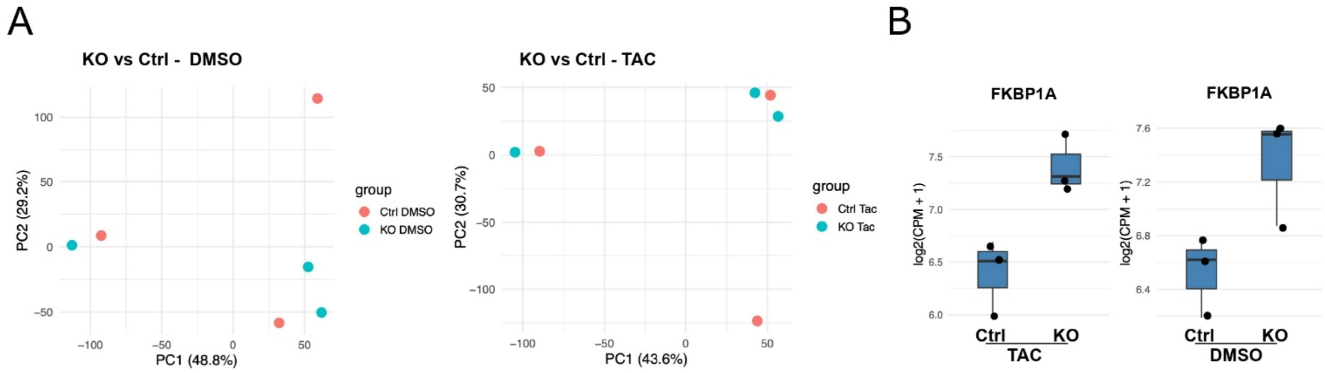

Supplementary Figure 7. Similar transcriptome between FKBP12-KO and control Tregs

(A) PCA of RNA-seq data (n=3) comparing KO vs Ctrl Tregs in DMSO (left) or Tacrolimus (right).
(B) FKBP1A RNA expression (logCPM) in KO vs Ctrl under both Tacrolimus and DMSO.

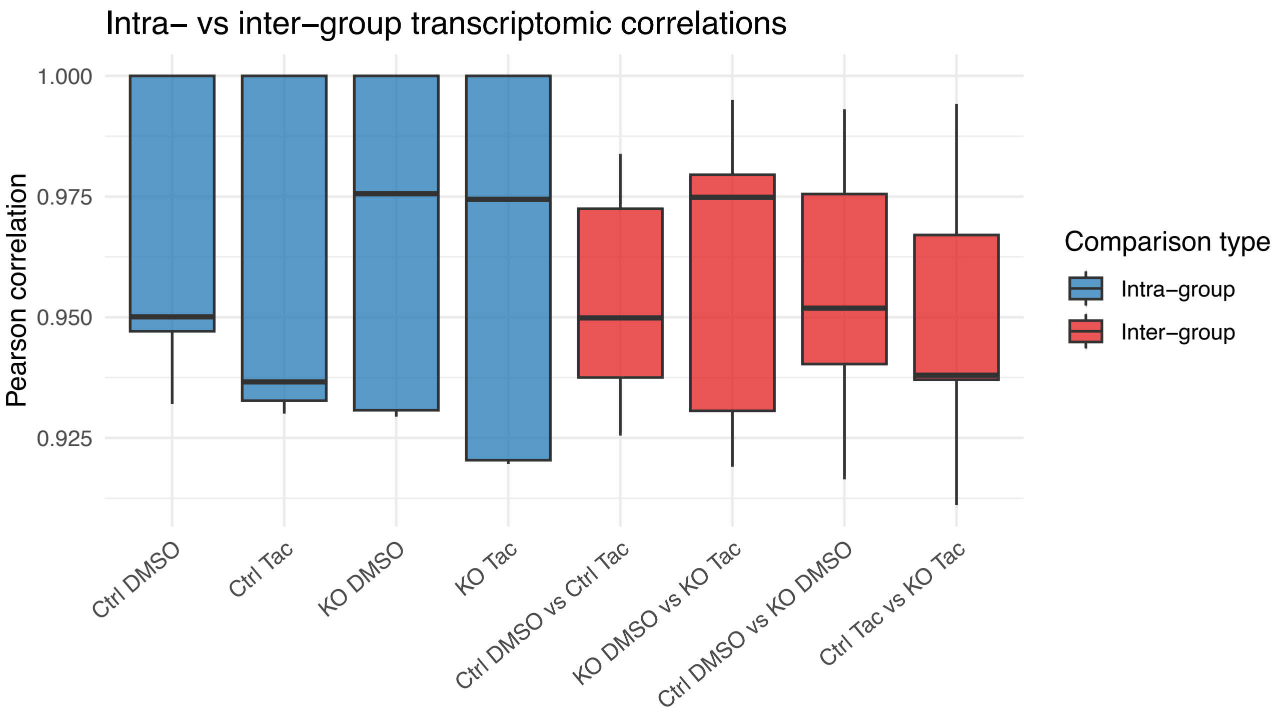

Supplementary Figure 8. Intra- and inter-group transcriptomic correlations across experimental conditions.

Pearson correlation coefficients were calculated from global RNA-seq gene expression profiles to assess similarity within and between experimental groups. Intra-group correlations (blue) reflect pairwise correlations among biological replicates within the same condition, whereas inter-group correlations (red) reflect pairwise correlations between FKBP12-KO and control Tregs across treatment conditions. Box plots display the median (center line), interquartile range (box), and range (whiskers). Intra-group correlations were consistently high, indicating strong reproducibility across replicates. Inter-group correlations were of comparable magnitude and largely overlapped with intra-group distributions, consistent with the absence of large-scale transcriptomic divergence between conditions.

1.2 Supplementary Tables

Buffer A		Buffer B	
**Products**	**Final concentration**	**Products**	**Final concentration**
HEPES pH7.8	10 mM	HEPES pH 7.8	10 mM
$MgCl_2$	8 mM	$MgCl_2$	8 mM
Sucrose	320 mM	Sucrose	320 mM
Triton-X 100	0.1% (v/v)	Triton-X 100	**NA**
Protease Inhibitor without EDTA	1 x	Protease Inhibitor without EDTA	1 x
H_2O mol		H_2O mol	

Supplementary Table 1: Nuclei extraction buffers composition.

Target	Clone	Dye	Compagnie
**Treg Sorting**			
CD4	RPA-T4	PE-cy7	Biolegend
CD25	M-A251	FITC	Biolegend
CD127	eBioRDR5	SB645	Thermo Fisher S.
Live-dead	Fixable Viability Dye	APC-efluor780	eBioscience™
**Treg phenotype analysis - A**			
CD4	SK3	Percp Cy5.5	BD Biosciences
CD25	M-A251	FITC	BioLegend
PD1	EH12.1	BUV737	BD Biosciences
LAG-3	11C3C65	BV785	Biolegend
TIM-3	F38-2E2	PE-Dazzle 594	Biolegend
Ki-67	B56	BV421	BD Biosciences
Foxp3	236A/E7	PE-cy7	Thermo Fisher S.
Live-dead	Fixable Viability Dye	APC-efluor780	eBioscience™
**Treg phenotype analysis - B**			
CD4	SK3	BUV737	BD Biosciences
TCR	IP26	FITC	Biolegend
CD62L	DREG-56	BV650	Biolegend
CD45RO	UCHL1	BUV395	BD Biosciences
Foxp3	236A/E7	PE-cy7	Thermo Fisher S.
Live-dead	Fixable Viability Dye	APC-efluor780	eBioscience™
**Treg phenotype analysis - C**			
CD4	SK3	BUV737	BD Biosciences
CCR2	REA624	Vio Bright FITC	Miltenyi Biotec
CCR4	L291H4	BV421	Biolegend
CXRC3	1C6/CXCR3	RB744	Biolegend
Foxp3	150D	PE	Thermo Fisher S.
Helios	22F6	APC	Biolegend
ICOS	C398.4A	BV510	BD Biosciences
CD39	A1	BUV395	BD Biosciences
CD73	AD2	BV605	Biolegend
CTLA-4	BNI3	BV786	BD Biosciences
LAP	S20006A	PE-cy7	Thermo Fisher S.
GARP	7B11	BV711	BD Biosciences
Live-dead	Fixable Viability Dye	ViaDye Red	Cytek Biosciences
**Intranuclear NFAT-staining**			
NFAT-1	D43B1	AF 488	Cell Signaling Technology
**Suppression Assay**			
CD3	UCHT1	BB515	BD Biosciences
CD4	SK3	BUV737	BD Biosciences
CD8	HIT8α	PE	Biolegend
PBMC	CDP	eF540	Thermo Fisher S.
Treg	CDP	eF670	Thermo Fisher S.
Live-dead	Fixable Viability Dye	APC-efluor780	eBioscience

Supplementary Table 2. Antibodies and dyes list.

Off-target site (sequence)	Mismatches	Chromosome	Cut site	PAM	Gene
GGGCGCACCTGCCCCCTGCG	3	chr1	3,690,316	CGG	TP73
GGGCGCCCCTTCCCCGGGCG	3	chr6	44,270,906	CGG	TMEM151B
GGGAGCACCGTCCCCCAGCG	3	chr17	19,743,186	TGG	ALDH3A1
GGGCGCACCTTCCCAAATTG	3	chr6	52,062,710	GGG	PKHD1
GGGAGCAGCTTCCCCAAGTG	3	chr1	17,232,971	AGG	PADI1
GAGGGCACCTTCCCCTAGCG	3	chr4	7,423,139	TGG	SORCS2
GGGCGCATCTTACCCAAACT	4	chr16	54,896,148	GGG	CRNDE
GGGACCACCTTCCCCAAGGA	4	chr5	176,597,717	TGG	GPRIN1
GGGCACACCTGCCCCGAGGG	4	chr18	79,874,410	GGG	KCNG2
GGGCGCAGCTGCGCCAGGCG	4	chr19	1,852,149	TGG	-
GGGCGGACTTTCCCAAAGCT	4	chr1	212,417,629	AGG	-
GGGGGCCCCTTCCCCAGGAG	4	chr9	128,422,007	AGG	CERCAM
GGGGGCACCTTCCCCTGGCA	4	chr17	76,147,653	AGG	RNF157-AS1
TGGCTCACCTTCCCCAACCC	4	chr17	81,180,364	TGG	AATK-AS1
GGGCGCGCCTTCACCTAGCC	4	chr11	34,160,251	TGG	ABTB2
GGGGGCACTTTCCCCCCAGCT	4	chr15	90,221,555	TGG	SEMA4B
GGGGGCACCCTCCTCAGGCG	4	chr10	103,564,285	AGG	NEURL1
AGGCCCACCTTCCCCAGGTG	4	chr14	73,262,848	AGG	PAPLN
GGGCTCACCTTCCCCATAGG	4	chr8	144,679,289	AGG	-
GCAGGCACCTTCCCCAGGCG	4	chr1	1,243,614	GGG	C1QTNF12

Supplementary Table 3. Off-target sgRNA analysis

In-silico off-target prediction for the sgRNA (5'-GGGCGCACCTTCCCCAAGCG-3') was performed using the Synthego Off-Target Analysis Tool V1.3, which identifies human genomic loci potentially susceptible to off-target cleavage by the selected sgRNA. No off-target sites with an identical sequence were detected, and only a limited number of sites with 3 mismatches were predicted.
